# *Moringa oleifera* as a multifunctional feed additive: synergistic nutritional and immunomodulatory mechanisms in livestock production

**DOI:** 10.3389/fnut.2025.1615349

**Published:** 2025-06-20

**Authors:** Raza Mohai Ud Din, Salwa Eman, Muhammad Hammad Zafar, Zhang Chong, Ahmed A. Saleh, Hosameldeen Mohamed Husien, Mengzhi Wang

**Affiliations:** ^1^Laboratory of Metabolic Manipulation of Herbivorous Animal Nutrition, College of Animal Science and Technology, Yangzhou University, Yangzhou, Jiangsu, China; ^2^College of Animal Science and Technology, Yangzhou University, Yangzhou, Jiangsu, China; ^3^Department of Animal and Fish Production, Faculty of Agriculture (Al-Shatby), Alexandria University, Alexandria, Egypt; ^4^College of Veterinary Medicine, Albutana University, Rufaa, Sudan; ^5^State Key Laboratory of Sheep Genetic Improvement and Healthy Production, Xinjiang Academy of Agricultural Reclamation Sciences, Shihezi, Xinjiang, China

**Keywords:** *Moringa oleifera*, nutritional profile, immunomodulatory mechanism, animal, feed additive

## Abstract

Investigating *Moringa oleifera (M. oleifera)*’ is potential as a livestock feed additive, this review explores its nutritional and phytochemical profiles and its mechanistic roles, specifically focusing on its immunomodulatory and antioxidant properties. *M. oleifera* is a rich source of diverse bioactive compounds, including polyphenols, alkaloids, terpenoids, flavonoids (e.g., quercetin, kaempferol), saponins, and tocopherols. These compounds exert significant immunomodulatory effects by modulating cytokine production and immune cell activity. Notably, *Moringa*-derived arabinogalactans (water-soluble polysaccharides comprising arabinose and galactose monomers) activate the gut-associated immune system through beneficial modulation of gut microbiota composition, increasing genera such as *Muribaculaceae* and *Lactobacillus*. The immunomodulatory activity is mediated via multiple pathways, including the promotion of anti-inflammatory cytokine secretion (e.g., IL-10) and the inhibition of pro-inflammatory enzymes [e.g., cyclooxygenase-2 (COX-2)]. Furthermore, *M. oleifera* exhibits potent antioxidant capabilities by enhancing endogenous defenses, neutralizing reactive oxygen species, and mitigating oxidative stress-induced tissue damage. These findings underscore *M. oleifera* is potential to enhance disease resistance and immune function in animals, advocating for its strategic incorporation into sustainable animal nutrition practices.

## Introduction

The entire livestock sector is facing unprecedented threats due to rising global consumption of animal-derived products like meat, milk, and eggs. Looking at the current situation of animal global meat production, there is a 55 percent increase between 2000 and 2022, reaching a total of 361 million tons (1). A key development during this period was the rise of chicken, which accounted for the largest share of this growth and surpassed pork as the most produced meat globally in 2022. Asia, home to nearly 60% of the world’s population, is an unparalleled force in global livestock production and consumption. The sheer demographic weight and ongoing economic growth within the continent position it as the primary driver of global livestock trends (2). China stands as the world’s largest livestock-producing country, a position that grants its internal dynamics significant influence over global markets and trends. Many publications have predicted the changes by 2050 regarding livestock production and consumption. By 2050, the demand for animal products globally is estimated to increase by 60 to 70%, and developing countries will have a bulk of this increase (3). Due to population growth, urbanization, and income growth, livestock, along with their products will change rapidly by 2050. While in animal health, welfare, and food security concerns, biotechnology and nanotechnology will play a key role (4). Poultry meat demand in sub-Saharan Africa is expected to rise by 214% by 2050, and for pork, by 161%, due to the main factor- urbanization, as well as demand for animal-sourced foods (5). China’s livestock industry change has severe global implications, especially large changes and effects expected in the 2050s (6). These changes have created an alarming situation regarding food security. Climate change affects livestock production and emission of greenhouse gases, hence the need to develop appropriate technologies for sustainable production and contribute to the global food supply (7). This growth requires improvement in feed supply technologies to increase productivity efficiently and cost-effectively.

Now the major inputs in livestock feed production are the traditional crops such as maize and soybean meals, which are the energy and source of protein, respectively, in animal feed. Farm animals account for over 30% of global food consumption, primarily relying on grains, with soybeans making up 90% of that total. A minimal amount of these grains is utilized within factory farming operations (8). However, the cultivation of these crops has its economic and environmental consequences. Soybean, for example, is one of the causes of deforestation more particularly in South America, and they also significantly contribute to greenhouse gas emissions (9). Soybean trade impacts the environment and socio-economy of the world, and therefore, there is a need to find ways to increase sustainability in the trade (10). Moreover, competition drawn from feeding humans and animals using these crops continues to complicate food security issues around the world (11, 12). The development of the “maize/soybean system” has taken place and changed the structure of competition and interaction between human and animal consumption of vegetable proteins (12).

Environmental sustainability is another pressing concern. Feed production currently accounts for a significant proportion of global agricultural land use, water consumption, and nitrogen pollution. Food systems are dependent on livestock greenhouse gas emissions, which can only be tackled on a global scale while supporting food security (13). The cultivation of soybean and maize, and exclusive for the feeding of stock rations, has been linked with undesirable effects on lands and water resources, planetary nutrient imbalances (14). These environmental impacts highlight the urgent need to identify and integrate alternative feed resources.

In addition to environmental challenges, economic volatility in feed prices poses significant issues for farmers. Grain prices in particular have trended higher in 2005 and 2006, which has put pressure on livestock feed costs and has also resulted in high volatility shocks (15). High and frequently changing feed costs, as well as high and rapidly changing output prices, are challenges to livestock farmers (16).

To meet the rising demand for livestock and poultry feed, researchers have sought alternatives to its traditional ingredients, which could be novel sources of protein and energy. Following recent years, innovations like the use of agricultural byproducts, insects, microalgae, and drought-resistant plants have increased. Tree leaves, along with traditional crops like *camelina* and oil seeds, offer promising alternative feed resources that can either replace or complement conventional crops in ruminant diets, leading to improved animal performance in a sustainable manner (17). Moreover, the livestock sector has identified insect-based products as a viable option to support sustainable development within the industry (18). Incorporating tree leaves as a feed ingredient presents a beneficial strategy, as they typically possess higher nutritional value than grasses, making them more appealing to herbivores (19).

**Figure 1:**
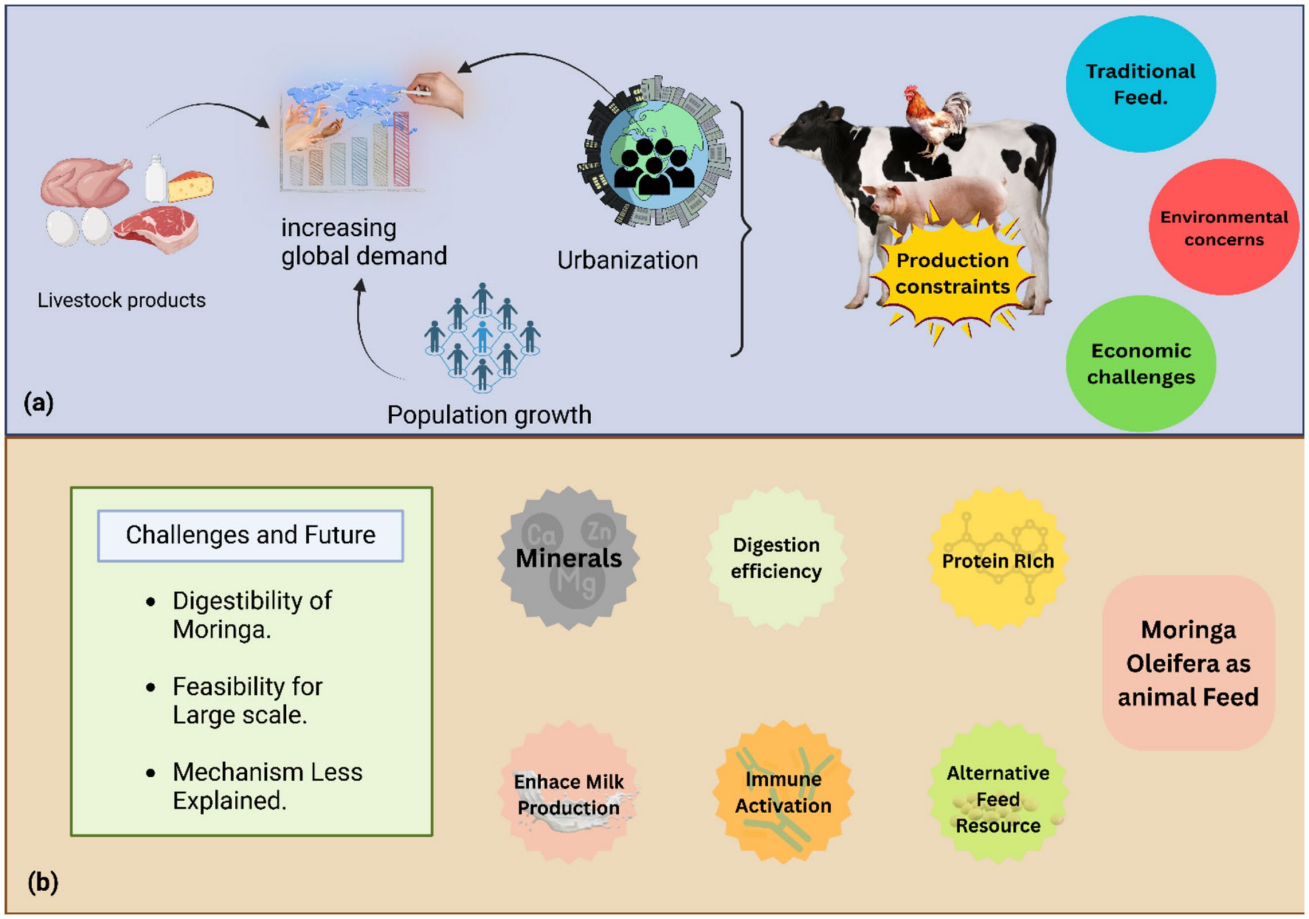
**(a)** This figure illustrates how increasing global demand for livestock products, driven by population growth and urbanization, creates operational difficulties for the worldwide livestock industry, including production constraints, reliance on traditional feed, environmental concerns from maize and soybean cultivation, and economic risks from variable feed costs. **(b)** This figure presents *M. oleifera* as a sustainable and alternative animal feed, highlighting its protein and mineral richness, and its potential to enhance digestion efficiency, activate immune responses, and increase milk production, thereby mitigating environmental damage and market fluctuations. The figure also identifies key research areas for *M. oleifera* ‘s effective implementation, including its digestibility, large-scale feasibility, and precise mechanisms of action.

A versatile plant, *Moringa oleifera (M. oleifera)*, with a lot of nutrients, can be used as an alternative feed and forage to traditional animal feed and fodder with no negative effects on health, survival, and reproduction (20). *M. oleifera* holds significant promise for addressing the livestock feeding crisis due to its rich nutrient content, elevated protein biological value, and positive effects on animal nutrition (8). Using *M. oleifera* leaves in place of sunflower seed cake for goat feed promotes dry matter consumption and improves product breakdown capabilities without losing nitrogen content (21). It contains phenolic and flavonoid compounds that have been associated with enhanced health, improved feed conversion efficiency, and better growth performance in livestock (20, 22). Due to its abundant nutrients, high protein biological value, good feeding effect, and great potential make *M. oleifera is* suitable mean to deal with the feeding crisis for livestock (8). The phenolics in *M. oleifera* leaves include a wide variety of kaempferol derivatives, caffeoylquinic acid, and feruloylquinic acid, and are responsible for their antioxidant capacity (23). Antioxidant potential in *M. oleifera* leaves appears very strong when combined with flavonoids, flavanols, phenolics, and proanthocyanidins elements (24). The direct radical scavenging action and indirect enhancement of cellular antioxidant defenses are expressed by antioxidant compounds in *M. oleifera* leaves. These substances completely remove free radicals while boosting antioxidant enzyme function, including superoxide dismutase and catalase, to lower oxidative stress levels (23, 25). Besides having antioxidant ability, *M. oleifera* leaves are rich in protein, minerals, vitamins, and essential amino acids (26). Analyses show *M. oleifera* leaves contain 28.7% crude protein alongside 7.1% fat, while the protein content exists as insoluble compounds that display poor *in vitro* digestibility (27). Ruminant farmers can prepare concentrated mixtures at 20% concentration, which improves goat performance while reducing methane releases (28). The introduction is explained graphically in Figure 1. Considering the importance of *M. oleifera*, in this review, we will give detailed nutritional composition of *M. oleifera* and the mechanism of action of its bioactive components as immunomodulator. Application of *M. oleifera* in animal feed is discussed in detail, along with challenges of *M. oleifera* as animal feed and future directions. This review systematically evaluates *M. oleifera* nutritional-phytochemical synergy and its translational potential for sustainable livestock production.

## Taxonomy, botanical characters, and cultivation

The initial description of *M. oleifera* was made in 1785 by the French naturalist Jean Baptiste Lamarck. The name “*M. oleifera* “is thought to originate from the Tamil word “murungai,” which translates to “twisted hand length structure of the young *M. oleifera* fruit.” In Latin, “oleum” signifies “oil,” while “ferre” means “to bear” (29). *M. oleifera* from the Moringaceae family represents a widely grown plant species that demonstrates significant medical characteristics as well as valuable nutritional benefits (30). Which has 13 species of different trees and shrubs, having the potential to be used for medicinal and nutritional purposes (31). All species have their native origin (Table 1).

**Table 1:** Species of the Moringaceae family with their native origin.

Species	Native region
*M. rivae*	Indigenous to Ethiopia and Kenya
*M. pygmaea*	Native to Somalia
*M. arborea*	Indigenous to Kenya
*M. borziana*	Native to Kenia and Somalia
*M. stenopetala*	Indigenous to Ethiopia and Kenya
*M. ovalifolia*	Indigenous to Angola and Namibia
*M. longituba*	Indigenous to Somalia, Kenya, and Ethiopia
*M. ruspoliana*	Native to Ethiopia
*M. peregrine*	Indigenous to the Horn of Africa and the Red Sea
*M. drouhardii, M. hildebrandi*	Indigenous to Madagascar
*M. concanensis*	Indigenous to the sub-Himalayan tracts of Northern
*M. oleifera lam*	Native to northwestern India and northeastern Pakistan

Among these, *M. oleifera* stands out as the most economically important variety that grows throughout Asia and is spreading across Africa and America (31, 32). It is a widely planted tree because it possesses high nutritional value while offering prospects to fight malnutrition (33).

*M. oleifera* is considered to have high phytonutrient content, with the ability of drought, is used to deal with malnutrition, and also has nutraceutical properties (34). It is a perennial tree with a height range from 5 to 12 cm (35).

*M. oleifera* thrives in tropical and subtropical regions, especially in areas with average annual rainfall between 1,000 and 2,000 mm and high levels of solar radiation (36). It has various climatic adaptations, including temperate, subtropical, and tropical regions, which makes it suitable for the semi-arid regions as a contributor to nutritional security (37). High vigor and resistance to salinity stress are promoted more effectively when seeds of *M. oleifera* are pre-soaked for 24 h, making them suitable for planting in areas that are subjected to salinity (38). *M. oleifera* is a high-yielder in terms of biomass production. The annual biomass yield of *M. oleifera* is reported as 43 to 115 t/h (39). The leaf production is 1–5 kg per tree annually, which is equal to10,000–50,000 kg/h if plants are cultivated at 1 m × 1 m spacing (40). Due to its distinctive cultivation attributes, *M. oleifera* is currently grown in India, southern China, and certain regions of Africa (41).

## Nutritional and phytochemical profile

*M. oleifera* is a superior nutritional source because of its extensive nutritional benefits, which make it an important supplement for livestock feed. *M. oleifera* demonstrates remarkable potential to solve livestock feeding problems through its supply of rich nutrients and excellent protein value, alongside positive nutritional advantages (8). Extensive research analysis has reported that *M. oleifera* leaves have great amounts of protein, vitamins, and amino acids, along with bioactive components that are beneficial to livestock. It offers valuable nutrient content as animal feed because it contains significant amounts of protein, along with carotenoids and minerals together with vitamins, and phytochemicals (42).

### Macronutrients and minerals

*M. oleifera* leaves are a rich source of essential macronutrients, providing building blocks for animal growth, development, and productivity. These macronutrients, including protein, lipids, and carbohydrates, significantly influence the overall nutritional value of *M. oleifera* as a feed resource. The leaves of *M. oleifera* contains 19.34–28.7%, providing a rich source of essential amino acids required for muscle growth and maintenance (27). Due to its high protein, *M. oleifera* is comparable with other conventional feed resources like maize in terms of percentage and with soybean, having a comparatively similar profile of amino acids (Table 2). The analysis of dietary fiber becomes essential for understanding plant cell wall nutritional value and its effects on animal digestive and absorption processes (43). *M. oleifera* leaves contain approximately 8.07% crude fiber, contributing to their overall nutritional profile (44). In general, the feed source with low fiber content is considered good due to better digestibility. Another appealing feature of *M. oleifera* is its content. In recent studies, the mineral content in the form of ash is reported to be 11.65% in *M. oleifera* leaves (45), which is significantly higher than soybean meal, which is 5.6–7.2 (46). Analysis of *M. oleifera* leaves cultivated in Gaborone, Botswana, revealed a variable ash content ranging from 5.6 to 9.1%, with a mean value of 7.34%. This variation underscores the influence of environmental factors and sample origin on the mineral composition of *M. oleifera* leaves (47). Another experiment testified to an ash content of 6.00% for *M. oleifera* leaf protein concentrate (48). The mineral composition of *M. oleifera* leaves exhibits variability, influenced by factors such as edaphic conditions and environmental parameters during cultivation. For instance, research has demonstrated comparable calcium concentrations in *M. oleifera* leaves (11,153 mg/kg) and roots (12,834 mg/kg), while seed calcium levels (565 mg/kg) were significantly lower. This observation highlights the division and difference accumulation of minerals within different plant tissues (49). There is a considerable amount of lipids in the leaves of *M. oleifera*, contributing to their nutritional value. On the dry meter basis, analysis of *M. oleifera* leaves samples have a range of lipid concentration from 1.7 to 10.42%. This variation is likely attributable to different factors, including the specific source of leaf sample, the analytical methods employed for lipid determination, and environmental conditions during cultivation (47). A mean lipid content of 7.8 ± 0.13% is shown by analysis of *M. oleifera* leaves from Botswana and Gaborone. This value exceeds previously reported values of 2.3, 5.2, and 3.0%, pointing out potential regional changes in *M. oleifera* lipid composition (50). The lipid fraction of *M. oleifera* leaves are characterized by the predominance of unsaturated fatty acids, notably palmitic, oleic, and linoleic acids (51). These purchases fulfill essential physiological roles and contribute to the energetic value of the leaves. A monounsaturated fatty acid, Oleic acid, has been implicated in the modulation of inflammatory responses and positive cardiovascular health outcomes (47).

**Table 2:** Proximate analysis: comparison of *M. oleifera* leaf powder with soybean meals and maize on a dry matter basis.

Nutrient	*M. oleifera* leaves (dry matter basis) g/100 g	Soybean meal (dry matter basis) g/100 g	Corn (maize) (dry matter basis) g/100 g
Crude protein	25–30	43.8–49.9	8–10
Crude fiber	8.07	5–7	2–3
Crude fat	2–5	18–20	3–4
Carbohydrate	40–45	35–40	70–75
ash	11.65	5–7	2–3
References	(163)	(8, 46)	(8, 164)

### Amino acids

Serving as the fundamental building blocks of proteins, amino acids are essential components in animal feed. Although they have a structural role, they also participate in cellular processes, including gene expression, cell signaling, and metabolic regulation. Therefore, that’s sufficient supply of amino acids is necessary for supporting overall well-being, optimal growth, development, reproduction, and lactation of animals (52). The metabolic pathways that are crucial for sporting growth, reproductive, and lactation functions are organized by amino acids, so in that way contributing to enhanced animal health (53). *M. oleifera*, due to its amino acid profile, is presented as a primary amino acid supplement in animal feed formulations, specifically when integrated with conventional forages (8). Phytochemical analyses have revealed the presence of 16–19 amino acids in *M. oleifera*, encompassing all ten essential amino acids: threonine, tyrosine, methionine, valine, phenylalanine, isoleucine, leucine, histidine, lysine, and tryptophan (54). *M. oleifera* exhibits comparatively elevated levels of lysine, leucine, histidine, glutamic acid, valine, isoleucine, alanine, phenylalanine, and arginine relative to other woody plant species (55). Some recent studies have reported *M. oleifera* is a notable source of essential amino acids, including threonine, valine, methionine, leucine, isoleucine, phenylalanine, histidine, lysine, and arginine (56, 57). Amino acids that cannot be synthesized *de novo* by animals are classified as essential. Efficient protein synthesis depends on the availability of both essential and non-essential amino acids at the ribosomal site, in proportions commensurate with the animal’s physiological needs. A deficit in any single amino acid can constrain the utilization of other amino acids within the dietary protein. The amino acid that initially restricts the rate of protein synthesis is defined as the first limiting amino acid. The second limiting amino acid, representing the next most deficient amino acid, can also negatively impact growth even when the first limiting amino acid is supplemented (58). For instance, in weaned calves consuming a corn and soybean meal-based diet, methionine has been identified as the first limiting amino acid, with lysine subsequently becoming limiting (59). Researchers have found that Supplementation with synthetic methionine or methionine-rich feed ingredients can improve dietary amino acid balance and consequently enhance animal performance (60, 61). Maintaining an appropriate balance between essential and non-essential amino acids in animal feed formulations is vital for ensuring optimal nutrition. As indicated in Table 3, *M. oleifera* leaves provide a variety of amino acids, with essential amino acids making up over 50% of the total amino acid content. Although the methionine content in *M. oleifera* leaves exceeds that of corn meal, it remains about two-thirds of the amount found in soybean meal (Table 3). Sulfur-containing amino acids are crucial for preserving cellular integrity and may also contribute to the detoxification of heavy metals through chelation (62). Dietary supplementation of cystine, a sulfur-containing amino acid, may be necessary in *M. oleifera* leaf meal formulations to ensure that animal requirements for sulfur-containing amino acids are met.

**Table 3:** Comparison: amino acid profile (g/100 g dry weight) of *M. oleifera* with conventional feed resources.

Amino acids	*M. oleifera* leaves (g/100 g)	Soybean meals (g/100 g)	Corn meal (g/100 g)
Essential			
Leucine	1.96	2.75	–
Lysine	1.637	2.43	0.22
Valine	1.413	1.70	0.26
Isoleucine	1.177	1.57	0.26
Phenylalanine	1.64	1.79	0.31
Methionine	0.297	0.60	0.43
Cystine	0.01	0.62	0.34
Tryptophan	0.486	0.64	1.03
Threonine	1.357	1.44	0.40
Non-essential			
Alanine	3.033	3.033	1.25
Aspartic Acid	1.43	1.43	1.97
Glutamic Acid	2.53	–	–
Glycine	1.533	2.048	–
Proline	1.203	–	–
Histidine	0.72	1.148	0.23
Serine	1.087	2.378	–
Tyrosine	2.65	1.53	0.08
Reference	(54)	(8, 165)	(8, 166)

### Mineral content

Ash content is often regarded as a measure of total mineral content. *M. oleifera* leaves serve as an important source of essential minerals, such as calcium, iron, potassium, and sodium (63). The levels of these minerals in *M. oleifera* leaves are generally higher compared to those found in other tree leaf species. Calcium ions are crucial for various cellular functions, including the regulation of cell motility, gene transcription, muscle contraction, and exocytosis (64, 65). *M. oleifera* leaves exhibit higher calcium content and bio accessibility compared to spinach and sweet potato leaves, suggesting their potential to enhance calcium intake, particularly in tropical and warm temperate regions (66). Notably, iron deficiency is frequently observed in many plant-based foods, except those derived from *M. oleifera* leaves. *M. oleifera* leaves provide substantially higher iron levels compared to other plant sources; for example, the iron content of *M. oleifera* leaves is reportedly 25 times greater than that of spinach (67). While *M. oleifera* leaves are a source of magnesium, which can positively influence milk yield and composition, for example, Magnesium supplementation in cattle diets has been reported to increase milk fat concentration and yield, with one study noting a 12% increase within 4 days (68). The recommendation to add excess magnesium salt to cattle feed is not universally supported. Although magnesium is essential for various physiological functions, including milk production, excessive intake can have detrimental effects. Cows do possess homeostatic mechanisms to regulate mineral balance, but their capacity to handle excess magnesium is limited. Hypermagnesemia, a condition characterized by elevated magnesium levels in the blood, can result from over-supplementation and lead to serious health problems, including muscle weakness, cardiac arrhythmias, and even death (69). Therefore, determining the appropriate magnesium level in cattle feed should be based on the animal’s growth stage, production status, and dietary context, rather than simply adding excess magnesium (Table 4).

**Table 4:** Comparison of mineral profile between *M. oliefra*, soybean, and maize.

Mineral	*M. oliefera* (mg/100 g)	Maize (mg/100 g)	Soybean (mg/100 g)
Ca	2016.5	10	310–1,593
K	1845	286	1,548–2,190
Na	8.13	15.9	–
Fe	19.37	2.3	58.22–172
Mg	322.5	139	280–580
Mn	1.6	–	41–193
Cr	0.02	–	–
Al	1.7	–	–
References	(163)	(164)	(167)

### Bioactive compounds and their distribution

*M. oleifera*, contributing to its recognized medicinal, nutritional, and therapeutic properties, presents a notable abundance of bioactive compounds (70). Bioactive compounds in *M. oleifera* include polyphenols, flavonoids, carotenoids, terpenoids, alkaloids, glucosinolates, tocopherols, and saponins. These compounds play important roles in enhancing animal health and optimizing livestock productivity (71). *M. oleifera* tree with its seeds, leaves, and bark packed with health boosting compounds like polyphenols, vitamins, and essential amino acids is considered a nutritional powerhouse (72). For example, glucosinolates and flavonoids play a crucial role as natural immune regulators, helping animals to combat infections and stress (73). Moreover, its saponins and tannins improve metabolic efficiency and nutrient absorption in animals, leading to healthier herds (74).

**Figure 2:**
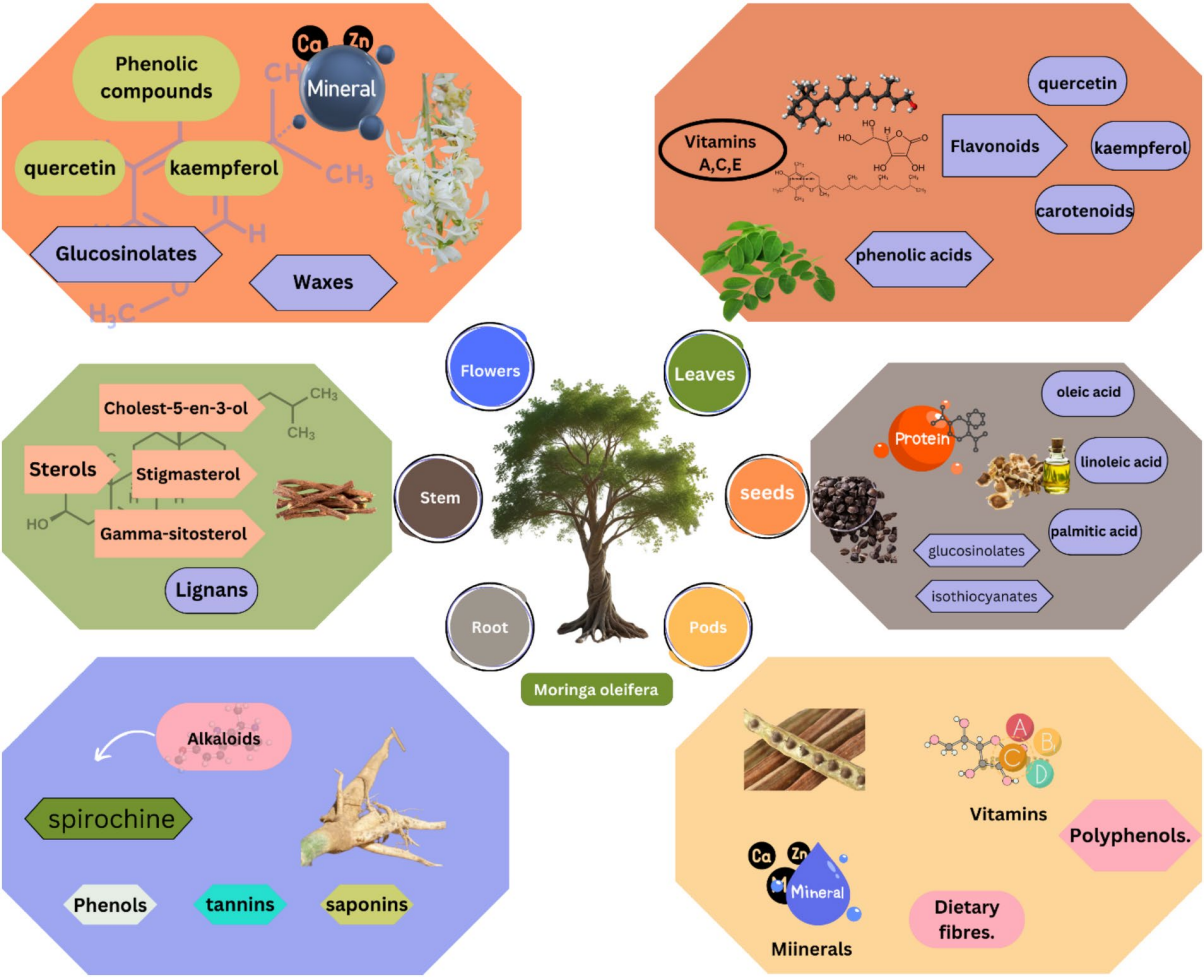
Spatial distribution of bioactive compounds in *M. oleifera* tissues. Leaves contain highest flavonoid concentrations (quercetin: 4.2 mg/g DW), while seeds are rich in isothiocyanates (4.1 mg/g DW). Pods provide unique combinations of fiber (32% DW) and immunomodulatory phenolics.

The leaves of *M. oleifera* are a rich source of nutrients, loaded with bioactive substances like phytosterols, polyphenols, and essential vitamins that actively reinforce livestock health. Flavonoids, such as kaempferol and quercetin, are among the most important immunomodulatory compounds found in leaves of *M. oleifera* (75). These compounds have been shown to influence the activity of immune cells and production of cytokines to modulate the cellular immune responses (76). *M. oleifera* is renowned for its diverse phytochemical composition. This includes a variety of polysaccharides contributing to their reported health benefits (77). Polysaccharides are complex carbohydrates made up of monosaccharide units, such as galactose, arabinose, rhamnose, glucose, and xylose (45). Among these, Arabinogalactan is identified as a significant polysaccharide found in *M. oleifera* leaves (78). These leaves are abundant in essential nutrients, including minerals, protein, and vitamins, as well as a diverse array of bioactive compounds such as saponins, tannins, and flavonoids (79). These elements take part in observed, antioxidant, anti-inflammatory, and immunomodulatory effects of plants (80, 81). *M. oleifera* pods are also a rich nutrient source and have bioactive components. They have protein, fiber, and immunoglobulin data, which are important components of immune function. Studies have shown that pods of *M. oleifera* can boost cell-mediated immunity in broiler chicken (82). Similarly, the seeds of *M. oleifera* are also a good source of oil and contain bioactive components like tannins and saponins. These cells can modulate immune response and have shown influence on immune cell activity (83). There is an enzyme in *M. oleifera* seeds called myrosinase, which can catalyze the production of isothiocyanates on plant damage or processing (84). The seeds of *M. oleifera* have shown anti-cancer activity by preventing cancer cell proliferation (85). They also have antimicrobial and anti-inflammatory activities (86). The detailed bioactive components in different parts of *M. oleifera* in graphically shown in Figure 2.

## Immunomodulatory mechanisms

### Gut microbiota modulation

Immunomodulation in livestock involves the targeted manipulation of the immune system to strengthen immune responses, ultimately leading to improved disease resistance and overall health (87). Dietary immunomodulation involves the incorporation of specific nutrients and bioactive compounds into animal feed to optimize immune function (88). For example, probiotics have demonstrated efficacy in improving livestock health by modulating gut microbiota composition and stimulating host immune responses through the secretion of specific factors and competitive exclusion of pathogenic bacteria (89). Likewise, the administration of immunomodulatory feed additives in cattle has been employed to modulate physiological parameters and enhance performance under stressful conditions, such as transportation (90). *M. oleifera*, commonly known as the “miracle tree,” is recognized for its immunomodulatory effects (91). These properties are attributed to its diverse array of bioactive compounds, including essential amino acids, oleic acid, vitamins, flavonoids, polyphenols, and minerals (92). *M. oleifera* contains flavonoids, like kaempferol and quercetin, that express significant anti-inflammatory activity (91). The activity of pro-inflammatory enzymes, such as cyclooxygenase and lipoxygenase is inhibited by these compounds to reduce the production of inflammatory mediators (93). This mechanism takes part in the reduction of inflammation and modulation of immune response in animals. There are different pathways for immunomodulation in animals, including Gut-associated immune system activation, antioxidant defense, and anti-inflammatory action. Comparison of bioactive compounds and immunomodulatory properties of *M. oleifera* with conventional feed crops (Supplementary Table S1).

### Gut-associated immune system activation

The gut, being the largest immunological organ, plays an important role both in digestion and nutrient absorption (94). The intestine of animals contains a vast and complex population of microorganisms, comprising billions of bacteria (95). These microbes play a critical role in nutrient absorption and digestion, taking part significantly in the body’s immune function and participating in a range of other biochemical and physiological processes (96). The composition of the intestinal microbiota of a healthy animal is predominantly composed of the phyla *Bacteroidetes, Firmicutes, Proteobacteria*, and *Actinobacteria*. *Bacteroidetes* and *Firmicutes* are particularly represented by the most abundant phyla, often covering 90% of the total intestinal microbial community (97). Any changes in intestinal flora can induce pathological changes within the intestinal tissue. Moreover, such disruption can contribute to the formation of carcinogenic compounds and chronic inflammation, thereby causing a significant risk to animal health (98).

Polysaccharides present in *M. oleifera* has been linked with different biological activities, including antioxidant properties, immunomodulatory effects, and potential antimicrobial actions (78). The diverse and significant biological activities of polysaccharides extracted from *M. oleifera* have been highlighted by recent researchers, leading to their increased prominence. Research teams have isolated and characterized MOP-1 as a newly discovered arabinogalactan that shows efficient *in vitro* antioxidant effects from *M. oleifera* leaves (99). Dong et al. (100) extracted MOP-2 from *M. oleifera* leaves and then examined the *in vitro* immunomodulatory activity.

**Figure 3:**
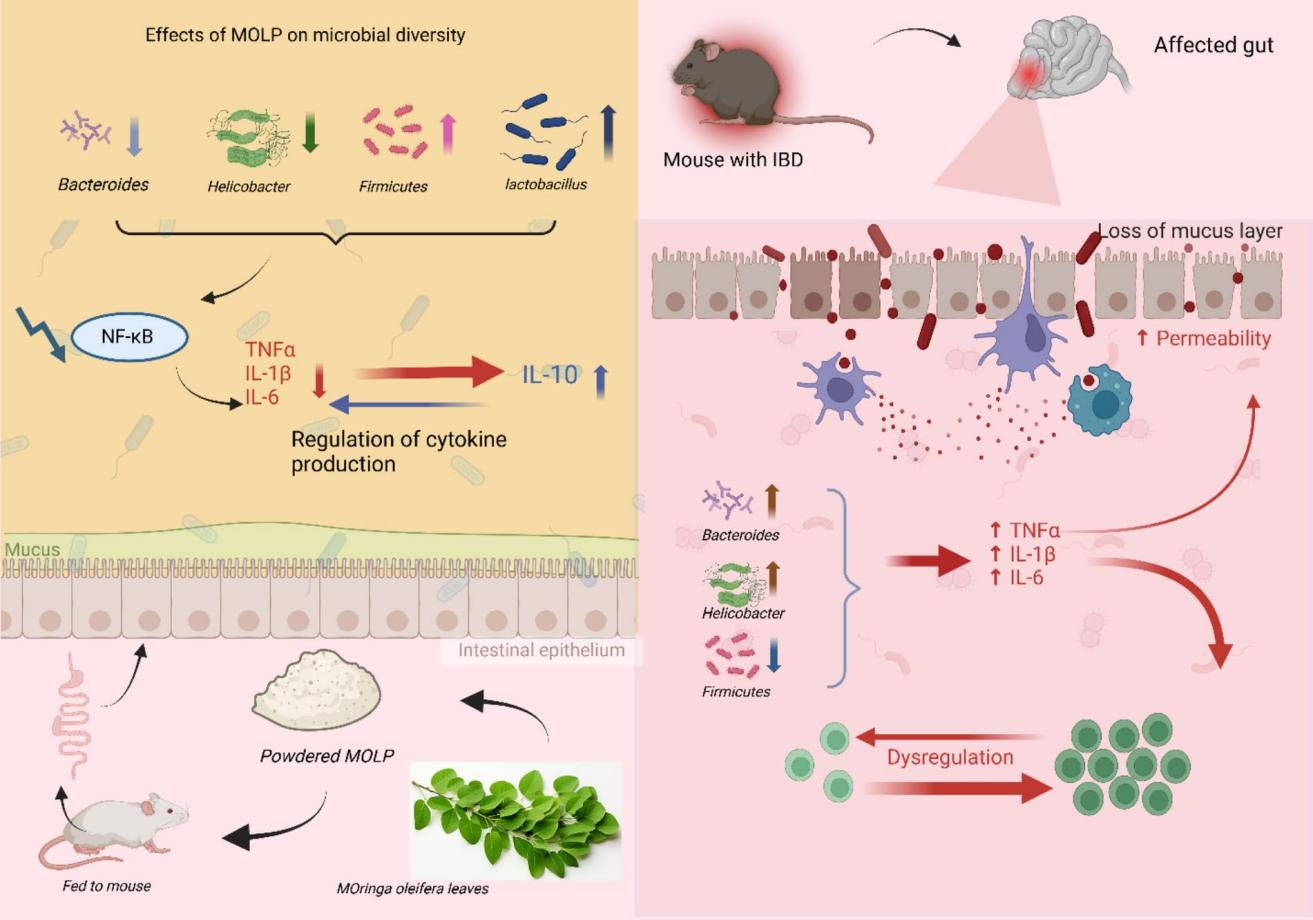
Explain the mechanism by which *M. oleifera* polysaccharides (MOLP) mitigate inflammatory bowel disease (IBD) involves modulation of gut microbiota composition and subsequent dampening of pro-inflammatory signaling. In IBD, dysbiosis is characterized by an increased abundance of pro-inflammatory bacteria (e.g., *Bacteroidetes* and *Helicobacter*) and a reduction in anti-inflammatory taxa (e.g., *Firmicutes*) contributes to mucosal barrier disruption and increased intestinal permeability. Oral administration of powdered *M. oleifera* leaves, rich in MOLP, appears to shift the gut microbial profile towards a more favorable composition, evidenced by increased beneficial genera such as *Lactobacillus* and the phylum *Firmicutes*. This modulation of the gut microbiota is associated with the downregulation of NF-κB signaling pathway and a consequent regulation of cytokine production, leading to a reduction in intestinal inflammation.

The immunomodulatory mechanism of *M. oleifera* leaf polysaccharides has been shown in recent studies. For instance, a study by Mohamed Husien et al. (78) has shown that high doses of *M. oleifera* polysaccharides promote intestinal health in UC mice by modulating gut microbiome compositions. The mechanism of action of *M. oleifera* in gut-associated immunomodulation is shown in Figure 3. Inflammatory Bowel Disease (IBD) is an inflammatory conditions that affect the gastrointestinal tract and can cause an increase in Bacteroides as reported previously (101). Treatment with MOLP-H significantly reduces *Bacteroidetes* abundance (29% decrease, *p* < 0.05) while increasing *Firmicutes* (40% rise) in DSS-induced colitis models (101). This observation aligns with a prior study that reported a 40% higher prevalence of *Firmicutes* compared to *Bacteroidetes* in mice subjected to a high-fat diet (102). *Bacteroidetes* and a few *Firmicutes* species, notably *Bacteroides* and *Lactobacillus*, have been implicated in modulating physiological conditions in mice subjected to dextran sodium sulfate (DSS) treatment (103). A decrease in *Lactobacillus* abundance has been correlated with an increase in ulcerative colitis (UC) induced by DSS. This suggests that *Lactobacillus* plays a beneficial role in immunomodulation (104). The study demonstrated that treatment with MOLP-H resulted in increased *Lactobacillus* levels (105). Research studies show that supplementation with *M. oleifera* leads to elevated *Lactobacillus* levels when obesity occurs through high-fat diet intake (104). Recent research exploring the interplay between gut microbiota and host immune responses has revealed that members of the Muribaculaceae family, a dominant component of the murine gut microbiota, can modulate host immunity. Natural killer (NK) cell activity is influenced by sucrose, while the nuclear factor-kappa B-alpha (NF-κB) signaling pathways experience impairment because of its presence (106). Treatment with MOLP induces changes in the gut microbiota composition, specifically increasing the abundance of families such as Muribaculaceae (107). These bacterial families have been identified to support greater activity of NK cells. The innate immune system relies on NK cells to perform recognition and elimination of abnormal infected cells (108). The pathogenic bacterium *Helicobacter* exists as an established cause of different gastric abnormalities. High *Helicobacter* counts in the body tend to worsen the outcomes of IBD (109). Research using mice established that *M. oleifera* polysaccharide administration minimized *Helicobacter* growth levels, while DSS treatment usually increases *Helicobacter* levels (105, 107).

### Anti-inflammatory pathways

The natural protective mechanism against stimuli is inflammation (110). The initial inflammatory response leads to acute inflammation, yet chronic inflammation occurs when the response endures for multiple weeks up to several years (111). Inflammation is essential for tissue regeneration and repair, necessitating optimal activation of both the innate and adaptive immune systems to mount an effective response to injury (112). Activated macrophages, key players in the inflammatory response, release a suite of pro-inflammatory cytokines, including tumor necrosis factor-alpha (TNF-*α*), interleukin-1 beta (IL-1β), interferon-gamma (IFN-*γ*), and interleukin-6 (IL-6) (113). These cells also generate reactive oxygen and nitrogen species, such as nitric oxide (NO), synthesized by inducible nitric oxide synthase (iNOS), contributing to the oxidative stress environment characteristic of inflammation (114).

Anti-inflammation, a process involving active suppression of pro-inflammatory signaling and restoration of tissue homeostasis, is crucial for limiting immunopathology following pathogen clearance or sterile injury. Chronic inflammation and subsequent tissue damage can be the result of failure to adequately control the inflammation cascade (115). The production of immunomodulatory molecules, like IL-10, and the regulation of specific signaling pathways to suppress the activity of pro-inflammatory responses can resolve the problem of inflammation, culminating in the repair of tissue homeostasis and preservation of immune equilibrium (116). This complex regulatory mechanism is crucial for maintaining physiological health (117). *M. oleifera* delivers anti-inflammatory benefits through its mix of health-enhancing chemical substances, including isothiocyanates, flavonoids, and phenolic acids. The anti-inflammatory effects of *M. oleifera* compounds are believed to be mediated through multiple mechanisms (93).

### Inhibition of pro-inflammatory enzymes

iNOS is an enzyme expressed in various immune cells, such as macrophages, in response to pro-inflammatory stimuli like lipopolysaccharide (LPS) and cytokines (118). Upon induction, iNOS catalyzes the conversion of L-arginine to NO. In contrast to the constitutive isoforms, endothelial NOS (eNOS) and neuronal NOS (nNOS), iNOS produces substantial quantities of NO over prolonged periods. Excessive NO production, especially in the presence of superoxide, can lead to the formation of peroxynitrite, a highly reactive species capable of damaging proteins, lipids, and DNA, thus contributing to the exacerbation of inflammation and tissue injury (119). The isothiocyanates, flavonoids, and phenolic acids present in *M. oleifera* can attenuate iNOS expression, thereby modulating NO production. This downregulation is often mediated through the suppression of upstream signaling pathways, particularly the NF-κB pathway (74). These bioactive compounds can stabilize the NF-κB inhibitor, IκB, preventing NF-κB translocation to the nucleus. Consequently, the transcriptional activity of the *iNOS* gene is diminished, resulting in reduced iNOS protein levels and a subsequent decrease in NO synthesis.

Similarly, Cyclooxygenase-2 (COX-2), an inducible enzyme, catalyzes the conversion of arachidonic acid to prostaglandins, such as prostaglandin E2 (PGE2) (120). These prostaglandins are pivotal inflammatory mediators implicated in the pathogenesis of pain, fever, and edema. In contrast to COX-1, which is constitutively expressed, COX-2 expression is markedly upregulated in response to inflammatory stimuli, positioning it as a key driver of inflammatory processes (121). The bioactive constituents of *M. oleifera* attenuates COX-2 expression, primarily through suppression of the NF-κB signaling pathway. Inhibition of NF-κB diminishes the transcriptional activity of the COX-2 gene, resulting in decreased COX-2 protein levels and a subsequent reduction in the synthesis of pro-inflammatory prostaglandins (93, 122). Furthermore, certain flavonoids present in *M. oleifera* may also exert a direct inhibitory effect on COX-2 enzyme activity.

### Regulation of cytokine production

Cytokines are a diverse group of signaling molecules that play a crucial role in the complex process of inflammation (123). Following tissue injury or pathogen invasion, immune cells, including macrophages, dendritic cells, and T lymphocytes, rapidly synthesize and secrete a variety of cytokines to initiate and regulate the inflammatory cascade (124). Pro-inflammatory cytokines, including TNF-*α*, IL-1β, and IL-6, are rapidly released at the site of tissue damage or infection (125). These cytokines initiate a cascade of events, activating resident cells and recruiting additional immune cells, such as neutrophils and monocytes, to the affected area, thereby amplifying and propagating the inflammatory response. It is very important to maintain a delicate equilibrium between pro-inflammatory and anti-inflammatory cytokines and prostaglandins. This equilibrium is essential for the development of targeted therapeutic strategies aimed at mitigating the detrimental effects of chronic inflammation and sepsis (126).

Administration of *M. oleifera bio actives*, such as *M. oleifera* isothiocyanate-1 (MIC-1) or MOLP, have demonstrated a significant decrease in tissue concentration and serum of pro-inflammatory cytokines, including TNF-*α*, IL-6, and IL-1β, in experimental models of acute inflammation or sepsis such as LPS-induced sepsis in mice (127). Moreover, along with suppression of pro-inflammatory signaling, certain *M. oleifera extracts* have shown the capacity to enhance the production of anti-inflammatory cytokines, such as IL-10, in animal models. This increase in IL-10 contributes to counterbalancing the inflammatory cascade and promoting resolution. For example, in a murine model of DSS-induced colitis, administration of MOLP not only reduced colonic levels of TNF-α and IL-1β but also concurrently elevated IL-10 expression, thereby facilitating the resolution of inflammation (78).

### Mechanism of action as an antioxidant

**Figure 4:**
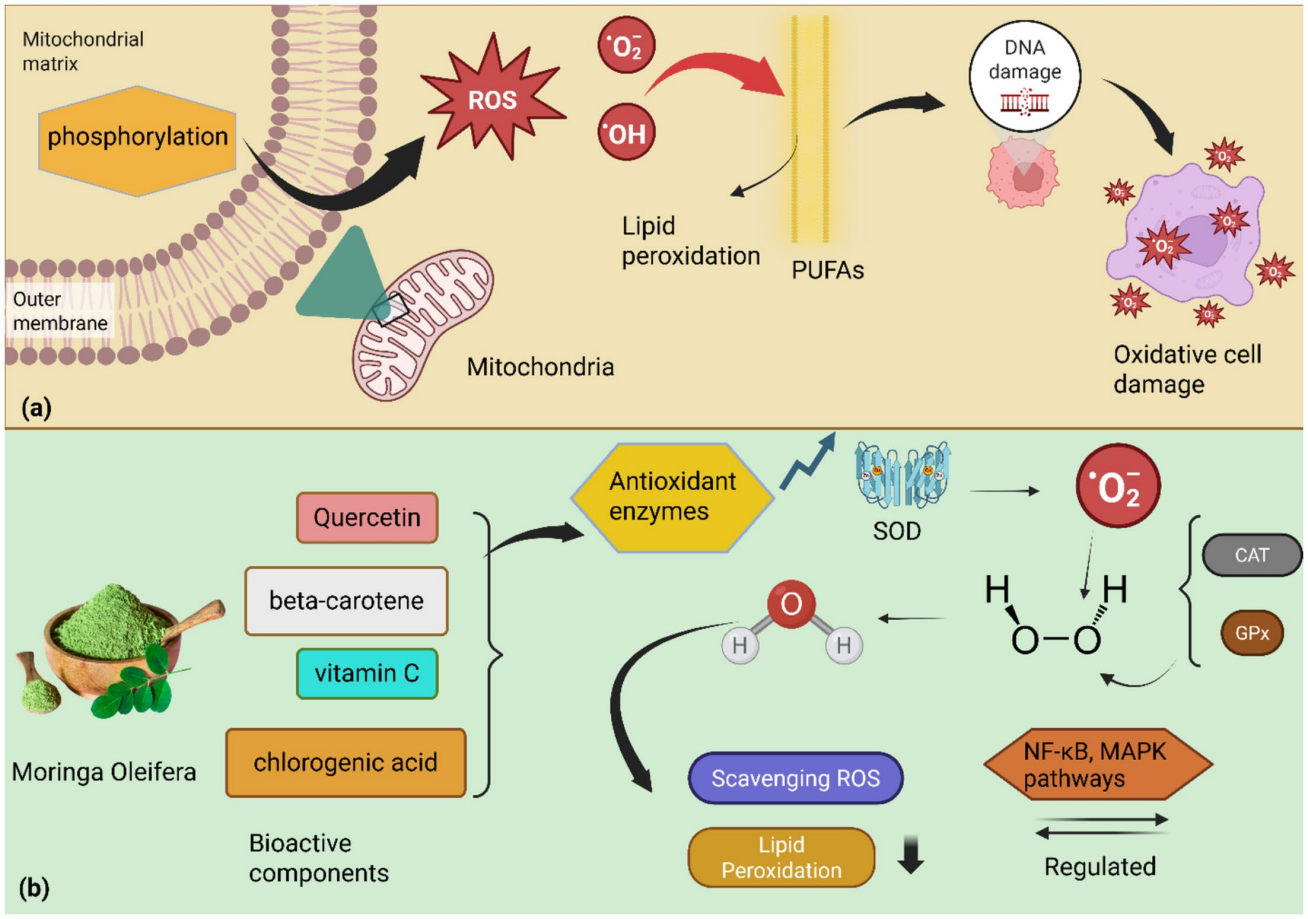
**(a)** Oxidative stress mechanism: mitochondrial oxidative phosphorylation generates superoxide radicals (O2•-) due to electron leakage from the electron transport chain. These radicals further transform into reactive oxygen species (ROS), including hydrogen peroxide (H_2_O_2_) and highly reactive hydroxyl radicals (OH•). When ROS production overwhelms cellular antioxidant defenses, it leads to an imbalance that attacks polyunsaturated fatty acids in cell membranes, initiating lipid peroxidation. Byproducts of lipid peroxidation subsequently cause DNA damage and overall oxidative cell damage. **(b)** *M. oleifera*’s antioxidant action: *M. oleifera* combats oxidative stress through its bioactive components, including quercetin, beta-carotene, vitamin C, and chlorogenic acid, which provide potent antioxidant protection. These antioxidants directly scavenge ROS and neutralize free radicals like superoxide (O2•-). *M. oleifera* also enhances the activity of key antioxidant enzymes such as superoxide dismutase (SOD), catalase (CAT), and glutathione peroxidase (GPx)1, enhancing cellular defense against oxidative damage. By scavenging ROS and boosting antioxidant enzyme activities, *M. oleifera* reduces lipid damage in membranes. Furthermore, *M. oleifera* modulates cellular signaling pathways, including NF-κB and MAPK, which regulate inflammatory responses triggered by oxidative stress, thereby ensuring cell health through its rigorous antioxidant properties.

Oxidative stress occurs when the generation of reactive oxygen species (ROS) overwhelms the body’s endogenous antioxidant defense mechanisms, leading to an imbalance that favors ROS accumulation and subsequent cellular damage (128). This imbalance can arise from either increased ROS production, decreased antioxidant capacity, or a combination of both (129). During oxidative phosphorylation, the process by which ATP is generated in mitochondria, electrons can escape from the electron transport chain, resulting in the formation of superoxide radicals (O₂^−^) (130). These superoxide radicals can subsequently be converted into other ROS, including hydrogen peroxide (H₂O₂) and highly reactive hydroxyl radicals (OḤ). While ROS plays essential physiological roles in cellular signaling and host defense, excessive ROS production overwhelms endogenous antioxidant systems, leading to a state of oxidative stress (131, 132). Activated immune cells, such as neutrophils and macrophages, generate ROS as a crucial component of the innate immune response against invading pathogens. While this ROS production is a physiological process essential for microbial killing, chronic inflammation can result in sustained and excessive ROS generation, which contributes to oxidative damage of host tissues and the progression of various diseases. Excessive production of ROS can lead to significant oxidative damage to critical cellular components, including lipids, proteins, and DNA (133). Such damage can impair cellular function, trigger cell death pathways (apoptosis or necrosis), and contribute to the aging process and the development of a wide range of pathological conditions (134). Oxidative stress can negatively impact immune function, rendering animals more susceptible to infections and disease (135). The mechanism of oxidative damage through phosphorylation is shown in Figure 4. In ruminants, for instance, oxidative stress has been linked to compromised immune responses, particularly during periods of physiological stress, such as the periparturient period or heat stress (136).

*M. oleifera leaves* are recognized for their significant antioxidant capacity, a property attributed to their rich composition of bioactive compounds (137, 138). These antioxidants play a critical role in scavenging free radicals and mitigating oxidative stress within biological systems (139). The abundance of bioactive compounds, including flavonoids, phenolic acids, and vitamin C, in *M. oleifera leaves* contributes significantly to their ability to mitigate oxidative damage and inflammation (140). Specific antioxidant constituents, such as quercetin, chlorogenic acid, and beta-carotene, present in *M. oleifera* contribute significantly to its protective effects against oxidative damage and inflammation (141, 142). *M. oleifera* has a series of events in its mechanism as an antioxidant, which are shown in Figure 4. These are scavenging free radicals, increasing endogenous antioxidant defenses, reducing lipid peroxidation, and modulating cellular signaling pathways that are related to oxidative stress (79). The 2,2-diphenyl-1-picrylhydrazyl (DPPH) assay is a widely used method to assess the activity of an antioxidant. Antioxidants reacted with that free radical DPPH by donating either an electron or a hydrogen atom (143). After the reaction, DPPH reduces to a non-radical form, α,α-diphenyl-*β*-picryl hydrazine, that results in loss of its purple color (144). Studies have shown that extracts of *M. oleifera have* a significant effect in reducing DPPH free radicals (145, 146). The radical scavenging and the antioxidant properties of different extracts of *M. oleifera* leaves from various agro-climatic regions were examined by Siddhuraju and Becker (147). The results found that the aqueous and aqueous ethanol extracts of freeze-dried leaves of *M. oleifera inhibit* 89.7–92.0% of peroxidation of linoleic acid and possess scavenging activities on superoxide radicals in the β-carotene-linoleic acid system. *M. oleifera* enhance the activity of endogenous antioxidant enzymes, in which glutathione peroxidase (GPx), catalase (CAT), and superoxide dismutase (SOD). This enhancement of these enzymes boosts the immune function against oxidative stress (148).

Free radicals interact with lipids in cell membranes, initiating a chain reaction of lipid peroxidation that can lead to cell damage and contribute to various diseases impacting the immune system (149, 150). Lipid peroxidation is recognized as a well-established biomarker of oxidative stress (151). This process primarily affects polyunsaturated fatty acids (PUFAs) such as linoleic acid, linolenic acid, and arachidonic acid, which are essential components of cell membranes (150, 152). The byproducts of this reaction include malondialdehyde (MDA) and 4-hydroxy-2-hexenal (153). These compounds possess mutagenic, cytotoxic, and neurotoxic properties, allowing them to alter DNA, damage cells, and harm nerve tissues (154).

*M. oleifera* has proven effective in inhibiting lipid peroxidation by lowering MDA levels. Research indicates that the percentage of lipid peroxidation observed in *M. oleifera* leaves and stems was 85.88 and 77.63%, respectively (155). In a study involving Swiss albino mice experiencing oxidative stress, pre-treatment with *M. oleifera leaf* extract successfully restored glutathione (GSH) levels, effectively reducing lipid peroxidation (156, 157). Similarly, a daily administration of *M. oleifera extract* for 60 days to rats with carbon tetrachloride (CCl4)-induced hepatic lipid peroxidation demonstrated a reduction in hepatotoxicity, attributed to phenolic compounds and flavonoids, including *β*-sitosterol, quercetin, and kaempferol found in the extract (158). In broiler chickens, supplementing *M. oleifera leaf* meal up to 5% of dry matter intake has shown improvements in fatty acid profiles and a reduction in lipid peroxidation (159). Recent studies on Nile tilapia (*Oreochromis niloticus*) have indicated that *M. oleifera* leaf extracts enhance feed utilization and growth while also improving the innate immune response, evidenced by increased lysosome levels and phagocytic activity (160, 161). A recent study involving crayfish (*Procambarus clarkii*) found that incorporating 1% of fermented *M. oleifera leaves* into the diet significantly improved growth performance and antioxidant capacity (162). Overall, due to its rich phenolic content, *M. oleifera exhibits* strong antioxidant properties.

## Future directions

Multiple essential investigations need completion before *M. oleifera* can achieve its complete potential as an environmentally friendly animal feed source for nutrition and health benefits. Research needs to focus on developing better processing methods that will boost the bioactive compound availability in *M. oleifera*. Scientific research is necessary to develop drying techniques and fermentation processes with enzymatic treatments, which will make *M. oleifera nutrients* more accessible after processing. Research needs to progress further to create well-balanced animal feed compositions that effectively integrate *M. oleifera into* animal dietary plans. There is a need to evaluate *M. oleifera* connection with dietary elements to discover optimal combinations that maximize nutrient uptake and promote animal development with improved wellness.

The ongoing research must focus on extended investigations to determine how *M. oleifera* supplementation affects livestock throughout multiple periods. More research about *M. oleifera* long-term influence on livestock performance must investigate its effects on reproductive outcomes and disease resistance, and animal welfare, regardless of demonstrated short-term enhancements in growth and feed conversion. The inconsistent bioactive component levels in *M. oleifera require* scientists to investigate the effects of growing conditions, together with the differences observed across cultivars. Extraction of *M. oleifera* cultivars alongside optimal cultivation methods that maximize beneficial compound concentrations will optimize the effectiveness of *M. oleifera* as a feed resource.

Research must determine the molecular processes that explain *M. oleifera* ability to modulate immune functions. The evaluation of *M. oleifera* bioactive compound-substance interactions with immune pathways and antioxidant systems, and gut microbiota needs comprehensive research to determine its potential as a natural animal feed immunostimulant. Research into *M. oleifera* biological mechanisms will optimize its value as a health promoter for livestock during times of stress.

Environmental assessment methods and economic modeling practices need to be implemented to determine the complete advantages *M. oleifera* has over conventional feed crops. Studies using life-cycle assessments need to evaluate the environmental effects of *M. oleifera farming* in addition to its capacity to minimize greenhouse gas emissions, water usage, and land degradation relative to maize and soybean as conventional feed crops. The cost-effectiveness assessment of *M. oleifera* in extended-scale livestock production requires economic studies to determine expense-to-worth ratios and the resulting lower feed costs while enhancing livestock health.

The rising acceptance of *M. oleifera as* an essential feed ingredient needs proper regulations to guarantee its secure usage within the livestock industry. Research about *M. oleifera* safety profiles alongside compliance with animal feed regulations will enable its integration into international commercial feeding systems. Driving market demand for sustainable livestock production with *M. oleifera will* be facilitated by educating consumers about its advantages. The successful adoption of *M. oleifera requires* research-based partnerships between researchers and both agricultural stakeholders and policymakers who will develop strategies for global livestock adoption.

*M. oleifera shows* extensive value because it represents a sustainable source of nourishing feed material for agricultural purposes. Research efforts into *M. oleifera must* advance through optimization of processing techniques, along with formulation research and studies of molecular effects and environmental and economic assessments. The implementation of these tactics ensures *M. oleifera significantly* contributes to solving current international issues regarding food supply stability and livestock health as well as environmental preservation.

## Conclusion

*M. oleifera* is increasingly recognized as a promising solution for sustainable livestock production due to its rich nutritional content and therapeutic properties. Packed with protein, essential amino acids, vitamins, and minerals, it enhances animal growth and overall health. Its immunomodulatory effects help regulate gut immunity and reduce oxidative stress, boosting disease resistance in livestock. Additionally, *M. oleifera serves* as a viable alternative to traditional feed sources like soybeans and maize, addressing concerns of feed supply, environmental impact, and price stability. Cultivating *M. oleifera in* arid regions can also contribute to food security in developing countries. While its potential is significant, the effective use of *M. oleifera requires* further research on diet optimization and its long-term effects on livestock performance. Overall, *M. oleifera offers* substantial benefits for enhancing livestock nutrition and promoting sustainable agricultural practices. *M. oleifera* triple-action benefits—nutritional (complete EAA profile), environmental (3.5 × lower carbon footprint than soybean), and therapeutic (40–60% cytokine reduction)—position it as a transformative feed additive. Critical findings include:

1 Optimal inclusion: 15% for ruminants (↑ milk yield 12%), 5% for poultry (↑ weight gain 8%).
2 Processing protocols: Freeze-drying retains 92% flavonoids vs. 67% in sun-drying.
3 Economic viability: 0.18/*kgproductioncostvs*0.31/kg for soybean meal.

Priority research areas:

- Long-term toxicity (>6 months consumption).
- Breed-specific formulation optimization.
- Policy frameworks for smallholder adoption.
