# Supplementary material for: Moringa oleifera as a multifunctional feed additive: synergistic nutritional and immunomodulatory mechanisms in livestock production
Source: Front Nutr. 2025 Jun 20;12:1615349. doi: 10.3389/fnut.2025.1615349 (PMC12226292; doi:10.3389/fnut.2025.1615349)
Supplement: Supplementary file 1 [file Table_1.pdf]

Table S1. Comparison of Bioactive Compounds and Immunomodulatory Properties of Moringa oleifera with Conventional Feed Crops

Bioactive Compound	Moringa oleifera	Soybean	Maize	Alfalfa	Refs
**Flavonoids**	- Quercetin, kaempferol (high concentrations).<br>Role: Inhibit pro-inflammatory enzymes (COX-2, iNOS), promote IL-10.	- Isoflavones (genistein, daidzein).<br>- Role: Mild anti-inflammatory effects .	- Limited flavonoids (e.g., luteolin).<br>- Role: Minimal immunomodulatory activity .	- Flavonols (quercetin derivatives).<br>- Role: Moderate antioxidant support .	1-8
**Polyphenols**	- Caffeoylquinic acid, feruloylquinic acid.<br>- **Role**: Scavenge ROS, reduce oxidative stress 6.	- Phenolic acids (ferulic acid).<br>- Role: Moderate antioxidant capacity .	- Ferulic acid (bound to fiber).<br>- Role: Limited bioavailability .	- Coumestrol, phenolic acids.<br>- Role: Antioxidant and phytoestrogenic effects .	
**Polysaccharides**	- Arabinogalactans (MOP-1, MOP-2).<br>- **Role**: Modulate gut microbiota (↑ *Lactobacillus*) 6.	- Oligosaccharides (raffinose, stachyose).<br>- Role: Prebiotic effects .	- Starch-derived polysaccharides.<br>- Role: Energy source .	- Pectin, fructans.<br>- Role: Mild prebiotic activity .	
**Saponins**	- High content.<br>- **Role**: Antimicrobial activity, immune stimulation 6.	- Soyasaponins.<br>- Role: Limited immune stimulation .	- Absent or trace amounts.<br>- Role: No significant contribution .	- Medicago-derived saponins.<br>- Role: Anti-parasitic .	
**Vitamins**	- Vitamin C, E, A.<br>- **Role**: Boost antioxidant enzymes (SOD, CAT) 6.	- Vitamin E (tocopherols).<br>- Role: Antioxidant protection .	- Vitamin B complex.<br>- Role: Metabolic support .	- Vitamin K, folate.<br>- Role: Blood health .	
**Minerals**	- Calcium (2,016 mg/100g), iron (19.7 mg/100g).<br>- **Role**: Reduce oxidative stress 6.	- Iron (8.2 mg/100g), zinc.<br>- Role: Basic mineral support .	- Phosphorus, magnesium.<br>- Role: Structural roles .	- Calcium, magnesium.<br>- Role: Bone health .	
**Unique Components**	- Isothiocyanates (e.g., moringin).<br>- **Role**: Anti-cancer via NF-κB inhibition 6.	- Trypsin inhibitors.<br>- Role: Anti-nutritional if unprocessed .	- Zein (storage protein).<br>- Role: No immunomodulatory relevance .	- Phytoestrogens (coumestrol).<br>- Role: Hormonal modulation .	

Ref;

1. Neupane, S.P., Stagnati, L., Dell'Acqua, M. et al. Genetic basis of Fusarium ear rot resistance and productivity traits in a heterozygous multi-parent recombinant inbred intercross (RIX) maize population. BMC Plant Biol 25, 639 (2025). https://doi.org/10.1186/s12870-025-06684-7
2. Gao, Y. (2025). Polyphenols in different parts of Moringa oleifera Lam.: Composition, antioxidant and neuroprotective potential. Food Chemistry, 475, 143207. https://doi.org/10.1016/j.foodchem.2025.143207
3. Alowo, D., Olum, S., Mukisa, I.M. et al. Prebiotic potential of oligosaccharides extracted from improved Ugandan varieties of millet, sesame, soybean, and sorghum: enhancing probiotic growth and enteric pathogen inhibition. BMC Microbiol 25, 307 (2025). https://doi.org/10.1186/s12866-025-04028-x
4. Yagi S, Rahman MTA, Zengin G, Eyupoglu OE, Spina R, Grosjean J, Abdalla AMA, Laurain-Mattar D. Phytoconstituents, antioxidant and enzyme inhibition activities of oilseeds and cakes of four underutilized wild edible plants in Sudan. Food Chem. 2025 May 9;486:144670. doi: 10.1016/j.foodchem.2025.144670. Epub ahead of print. PMID: 40367824.
5. El-Tanbouly, R., Gaber, M.A., Omran, S. et al. Moringa (Moringa oleifera) green-synthesized copper oxide nanoparticles for the drought tolerance of tomato (Solanum lycopersicum). BMC Plant Biol 25, 685 (2025). https://doi.org/10.1186/s12870-025-06708-2
6. Tlahig, S., & Elfalleh, W. (2025). Alfalfa as a nutritional and functional food resource: Applications and health benefits. Food Bioscience, 68, 106762. https://doi.org/10.1016/j.fbio.2025.106762
7. Li, B., Fan, R., Sun, G. et al. Flavonoids improve drought tolerance of maize seedlings by regulating the homeostasis of reactive oxygen species. Plant Soil 461, 389–405 (2021). https://doi.org/10.1007/s11104-020-04814-8
8. Lin, P., Liu, S., Fu, Z., Luo, K., Li, Y., Peng, X., Yuan, X., Yang, L., Pu, T., Li, Y., Yong, T., & Yang, W. (2024). Rhizosphere flavonoids alleviate the inhibition of soybean nodulation caused by shading under maize-soybean strip intercropping. Journal of Integrative Agriculture. https://doi.org/10.1016/j.jia.2024.09.030
